# CMT: A Constrained Multi-Level Thresholding Approach for ChIP-Seq Data Analysis

**DOI:** 10.1371/journal.pone.0093873

**Published:** 2014-04-15

**Authors:** Iman Rezaeian, Luis Rueda

**Affiliations:** School of Computer Science, University of Windsor, Windsor, Ontario, Canada; Northwestern University, United States of America

## Abstract

Genome-wide profiling of DNA-binding proteins using ChIP-Seq has emerged as an alternative to ChIP-chip methods. ChIP-Seq technology offers many advantages over ChIP-chip arrays, including but not limited to less noise, higher resolution, and more coverage. Several algorithms have been developed to take advantage of these abilities and find enriched regions by analyzing ChIP-Seq data. However, the complexity of analyzing various patterns of ChIP-Seq signals still needs the development of new algorithms. Most current algorithms use various heuristics to detect regions accurately. However, despite how many formulations are available, it is still difficult to accurately determine individual peaks corresponding to each binding event. We developed Constrained Multi-level Thresholding (CMT), an algorithm used to detect enriched regions on ChIP-Seq data. CMT employs a constraint-based module that can target regions within a specific range. We show that CMT has higher accuracy in detecting enriched regions (peaks) by objectively assessing its performance relative to other previously proposed peak finders. This is shown by testing three algorithms on the well-known FoxA1 Data set, four transcription factors (with a total of six antibodies) for *Drosophila melanogaster* and the H3K4ac antibody dataset.

## Introduction

Determining the interaction between a protein and DNA to regulate gene expression is a very important step toward understanding many biological processes and disease states. ChIP-Seq is one of the techniques used for finding regions of interest in a specific protein that interacts with DNA [1]–[7]. The main process consists of Chromatin-immunoprecipitation (ChIP) followed by sequencing of the immuno-precipitated DNA with respect to the reference genome. In the first step, chromatin is isolated from cells or tissues and then fragmented. After pruning, the fragments are sequenced and aligned to the reference genome. These aligned fragments produce a histogram in such a way that the 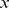-axis represents the genome coordinates and the 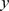-axis represents the frequency of the aligned fragments in each genome coordinate.

Detecting protein binding sites from large sequence-based datasets with millions of short reads represents a challenging bioinformatics problem that requires considerable computational resources, despite the availability of a wide range of tools for ChIP-chip data analysis [8]–[11]. The growing popularity of ChIP-Seq technology has increased the need to develop new algorithms for peak finding. Due to mapping challenges and biases in various aspects of the existing protocols, identifying relevant peaks is not a straightforward task.

Different approaches have been proposed for detecting peaks on ChIP-Seq mapped reads. Zhang *et al.* presented a *model-based analysis of ChIP-Seq data* (MACS), which analyzes the data generated by short read sequencers [12]. MACS models the length of the sequenced ChIP fragments and uses it to improve the spatial resolution of predicted binding sites. A two-pass strategy called *PeakSeq* has been presented in [13]. This strategy compensates for signals caused by open chromatin, as revealed by the inclusion of the controls. The first pass identifies putative binding sites and compensates for genomic variations in mapping the fragment sequences. The second pass filters out sites not significantly enriched compared to the normalized control, computing precise enrichments and significance of each detected peak. *Tree shape Peak Identification for ChIP-Seq* (T-PIC) is a statistical approach for calling peaks in ChIP-Seq data [14]. This approach is based on evaluating the significance of a robust statistical test that measures the extent of pile-up reads. Specifically, the shapes of putative peaks are defined and evaluated to differentiate between random and non-random fragment placements on the genome. Another algorithm for detecting relevant peaks is *site identification from paired-end sequencing* (SIPeS) [15], which can be used for identification of binding sites from short reads generated from paired-end Illumina ChIP-Seq technology. Qeseq is another method for analyzing the aligned sequence reads from ChIP-Seq data and identifying enriched regions [4]. The algorithm consists of three main modules: relative enrichment estimation, cluster detection and filtering possible artifacts. It cycles between its first two modules by removing detected clusters and evaluating enrichment in the rest of the signal. In the last step, a filter module is used to remove artifacts from the results.

One of the downsides of the existing methods is that they try to find all the enriched regions regardless of their length. These regions can be grouped by their length. For example, histone modification sites normally have a length of 50 to 60 kbp, while some other regions of interest such as exons have a much smaller length of around 100 bp. Using these methods, there is no way to focus on regions with a specific length and all of the relevant peaks should be detected first. This is a time consuming task that forces the model to process all possible regions. To deal with this issue, *constrained multi-level thresholding* (CMT) is proposed in this paper. Using CMT, we are able to search a specific region with a certain length which consequently increases the performance of the model. CMT is also able to target as many regions as the other methods simply by increasing the range for minimum and maximum lengths of the regions, which can be adjusted by the user based on their needs. The results of the experiments show that the proposed model is able to achieve a higher degree of accuracy than the previously proposed methods.

## Results

To evaluate the proposed model, we have used various datasets. The first dataset is *FoxA1* [12], which contains experiment and control samples of 24 chromosomes. The FoxA1 protein is known to cooperatively interact with estrogen receptor in breast cancer cells [16], [17]. We consider another six datasets which belong to four transcription factors (with a total of 6 antibodies) for *Drosophila melanogaster* using published data from the Eisen lab [18] (available at the NCBI GEO database [19], accession no. GSE20369). These four transcription factors, namely Hunchback (HB), Krppel (KR), Giant (GT) and Caudal (CAD), have been obtained by immunoprecipitating binding regions with affinity purified rabbit polyclonal antibodies raised against the *D. melanogaster* versions of the key A-P regulators. The other dataset is a genome-wide map of the *H3K4ac* antibody with ability to covalent acetylations in histones [20], which occur mainly at the N-terminal tails of the histone, and can affect transcription of genes.

**Figure 1:**
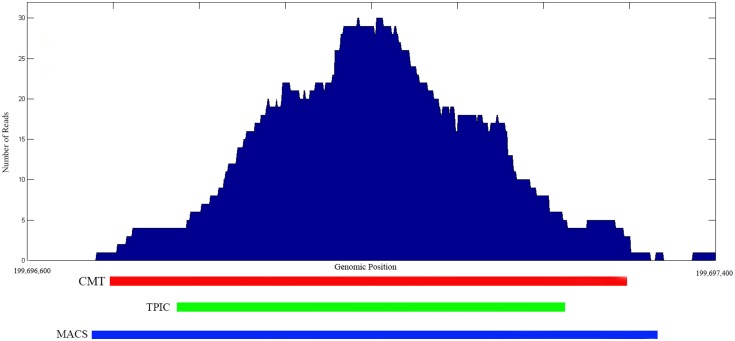
A detected region from the FoxA1 dataset for chromosome 1. The 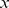-axis corresponds to the genome position in bp and the 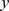-axis corresponds to the number of reads.

As in [14], the experiment and control histograms were generated separately by extending each mapped position (read) into an appropriately oriented fragment, and then joining the fragments based on their genome coordinates. We compare CMT, MACS [12] and T-PIC [14]. Figure 1 shows a typical region detected in chromosome 1 by CMT, MACS and T-PIC along with the corresponding base pair coordinates in the FoxA1 dataset. As shown in the plot, all three methods found the position of the peak accurately.

Computing the enrichment score for each method proceeds as follows. Random intervals from the genome are created by selecting the same number of intervals with the same lengths from each chromosome as in the called peaks but with random starting locations. Then, the number of occurrences of the binding motif in the called peaks and the random intervals are counted. Table 1 shows the binding motifs corresponding to each dataset. The motifs for CAD, GT, HB, and KR datasets have been obtained from [21], while the binding motif for the FoxA1 dataset has been obtained from [22]. The enrichment score is the ratio of the number of occurrences in the called peaks divided by the number of occurrences at random intervals.

**Table 1:** Binding motifs corresponding to each dataset.

FoxA1	CAD	GT	HB	KR
TGCATG	TTTATTG, TTTATGA	TTACGTAA	TTTTTT	GANGGGT, AANGGGT

### Comparison with Other Methods

**Figure 2:**
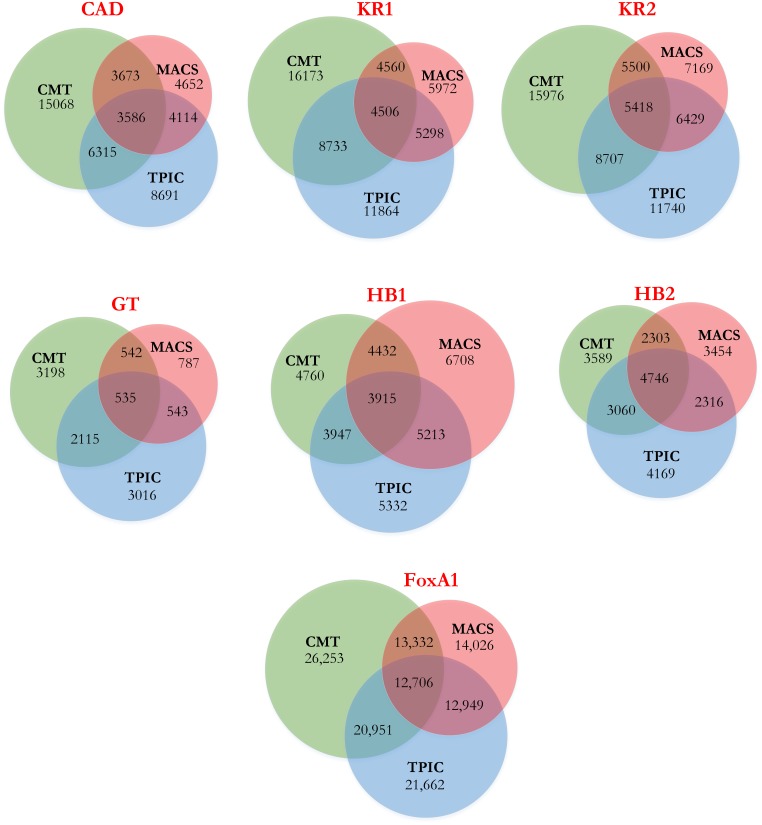
Venn diagrams corresponding to all datasets. Each Venn diagram shows the number of detected regions by CMT, MACS and T-PIC in each dataset along with the number of detected regions by each pair and all aformentioned methods.

Figure 2 shows the Venn diagram corresponding to each dataset for all three methods. We consider a peak detected by two methods to be overlapping, if the summit of the peak is located in the detected region by both methods. For example, Figure 1 shows an overlapping region detected by all three methods. In the FoxA1, KR1 and KR2 datasets, the number of regions selected by CMT is relatively higher than those of the other methods. These regions have mostly a small footprint which has not been detected by T-PIC or MACS. In the GT dataset, the numbers of regions detected by CMT and T-PIC are comparable. Interestingly, MACS detected only one fourth of the peaks detected by two other methods. In the HB1 and HB2 datasets, the result is the opposite and MACS detects more regions than T-PIC and CMT. In the H3K4ac dataset, while the number of histone modification sites using CMT and T-PIC are comparable, we were not able to obtain any regions with minimum size of 2,000 bp using MACS even after various parameter adjustments. Also, Table 2 shows a summary of prediction for the proteins found by each method. Each value represents the percentage of peaks detected by each method which are also detected by the other methods. For example, CMT detects 95.1% of the peaks detected by MACS, while MACS only detects 50.8% of significant peaks detected by CMT in the FoxA1 dataset. This demonstrates the wide spectrum and specificity of the proposed CMT algorithm. As mentioned earlier, since MACS was not able to detect wide peaks in the H3K4ac dataset, the corresponding cells in Table 2 have been marked with *N/A* (not applicable).

**Table 2:** Percentage of common peaks detected by each method included in the comparison and related to each protein of interest.

		CMT	T-PIC	MACS
**FoxA1**	CMT	100	79.8	50.8
	T-PIC	96.7	100	59.8
	MACS	95.1	92.3	100
**CAD**	CMT	100	41.9	24.4
	T-PIC	72.7	100	47.3
	MACS	79.0	88.4	100
**GT**	CMT	100	66.1	16.9
	T-PIC	70.1	100	18.0
	MACS	68.9	69.0	100
**HB1**	CMT	100	82.9	93.1
	T-PIC	74.0	100	97.8
	MACS	66.1	77.7	100
**HB2**	CMT	100	85.3	64.2
	T-PIC	73.4	100	55.6
	MACS	66.7	67.1	100
**KR1**	CMT	100	54.0	28.2
	T-PIC	73.6	100	44.7
	MACS	76.4	88.7	100
**KR2**	CMT	100	54.5	34.4
	T-PIC	74.2	100	54.8
	MACS	76.7	89.6	100
**H3K4ac**	CMT	100	16.1	N/A
	T-PIC	16.7	100	N/A
	MACS	N/A	N/A	N/A

Table 3 shows a comparison between the three peak finding algorithms considered in this study. As shown in the table, in terms of enrichment ratio, CMT is the best among these methods, overall. The difference between CMT, w.r.t. MACS and T-PIC is considerable in some datasets such as GT, HB1 and HB2. On the other hand, the average size of the peaks is relatively smaller than those of the other two methods, which implies that CMT is able to detect significant peaks more precisely. This helps determine the actual footprint of a binding site accurately. We do not report the enrichment scores for the H3K4ac dataset, since the binding motifs for this dataset are not reported in [20]. In another comparison, using the FoxA1 dataset, we evaluate the enrichment score of peaks that have been detected by one of the methods and missed by the other two. Table 4 shows the average size and enrichment score of CMT, MACS and T-PIC.

**Table 3:** Peak number, length and score comparison.

Dataset	Method of Comparison	CMT	T-PIC	MACS
FoxA1	Mean length of peaks	277	303	373
	Enrichment ratio	2.39	2.42	1.83
CAD	Mean length of peaks	476	818	507
	Enrichment ratio	0.92	0.88	0.93
GT	Mean length of peaks	303	866	194
	Enrichment ratio	4.21	1.98	3.02
HB1	Mean length of peaks	365	920	429
	Enrichment ratio	2.03	1.57	1.80
HB2	Mean length of peaks	343	891	228
	Enrichment ratio	2.11	1.56	1.99
KR1	Mean length of peaks	517	728	492
	Enrichment ratio	1.91	1.83	1.95
KR2	Mean length of peaks	513	737	500
	Enrichment ratio	1.94	1.75	2.10

Comparison between CMT, MACS and T-PIC based on the number and mean length of detected peaks and enrichment score.

**Table 4:** Length and enrichement score comparison.

	CMT	T-PIC	MACS
Mean length of peaks	220	421	337
Enrichment ratio	2.74	2.92	1.67

Comparison between CMT, MACS and T-PIC the average length of detected peaks and enrichment score on the FoxA1 dataset.

A conceptual comparison of CMT and other peak finding methods based on their features is shown in Table 5. As shown in the table, different algorithms require different sets of parameters for processing the data, including 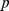-value, 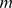-fold, window length, among others. CMT gives users the ability to fine tune the procedure based on their needs. Including the minimum and maximum range for regions of interest helps the procedure target regions within a specific range easily. It also boosts CMT to detect very small (or very large) regions, depending on the parameters settings, more than T-PIC and MACS, as shown in Figure 2, where most of the peaks have a small footprint. This makes the peak detection process rather difficult for other methods. CMT overcomes this problem by using the specified ranges for minimum and maximum size of the target regions and scan the histogram with more emphasis on peaks within the specified range.

**Table 5:** Conceptual comparison of recently proposed methods for finding peaks in ChIP-Seq data.

Method	Peak selection criteria	Peak ranking	Parameters
GLITR	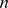 : Classification by height and relative enrichment	Peak height and fold enrichment	Target FDR, number of nearest neighbors for clustering
MACS	Local region Poisson 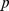 -value	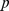 -value	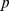 -value threshold, tag length, 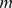 -fold for shift estimate
PeakSeq	Local region binomial 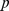 value	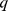 value	Target FDR
Quest v2.3	Height threshold, background ratio	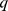 value	KDE bandwidth, peaks height, sub-peak valley depth, ratio to background
SICER v1.02	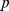 value from random background model, enrichment relative to control	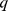 value	Window length, gap size, FDR (with control) or 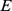 -Value (no control)
SiSSRs v1.4	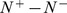 sign change, 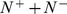 threshold in region	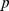 value	FDR, 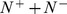 threshold
T-PIC	Local height threshold	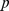 -value	average fragment length, significance 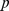 -value, minimum length of interval
Qeseq	Local enrichment significance	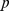 -value	no parameter
CMT	Height threshold and volume difference	fold enrichment	average fragment length, minimum and maximum region size, cut-off, minimum supported reads

**Figure 3:**
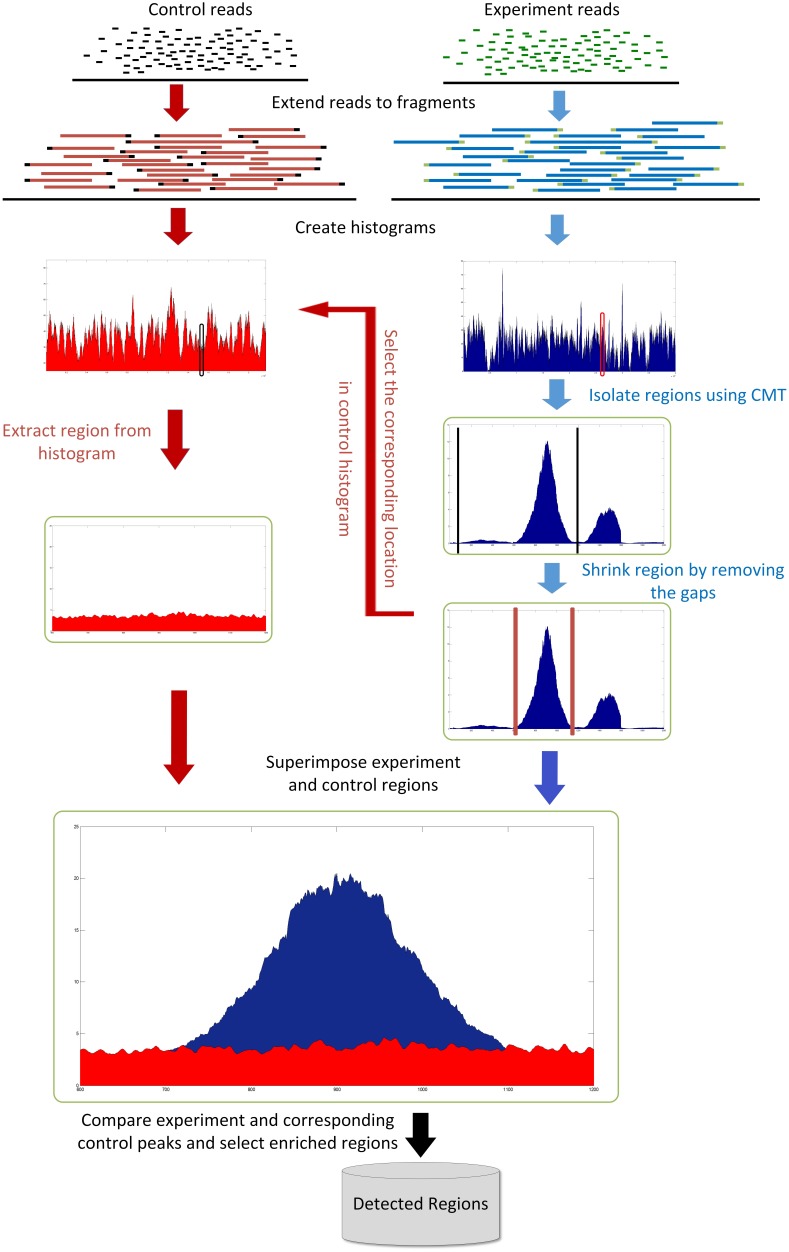
Schematic diagram of the pipeline for finding significant peaks.

To compare the prediction specificity of these three methods, we swapped the ChIP and control samples, and calculated the false discovery rate (FDR) of each of these methods as follows:
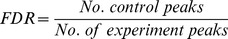
(1)For example, if we have 100 peaks selected and by swapping the experiment and control samples and using the same parameters we obtain 30 peaks, then the FDR would be 30%. Figure 3 shows the comparison between CMT, MACS and T-PIC on the FoxA1 dataset based on the FDR rate and the number of selected peaks. As shown in the figure, while CMT and MACS act similarly, T-PIC falls behind with its higher FDR rate. There is a clear advantage for CMT in finding the top 1,000 regions, while from the 1,000 to 10,000 top regions, MACS yields a slightly lower FDR rate. Due to possible background noise in the data and also because the regions are relatively small, CMT is able to find peaks with lower FDR than T-PIC and MACS when we target a small subset of regions with high enrichment level.

**Figure 4:**
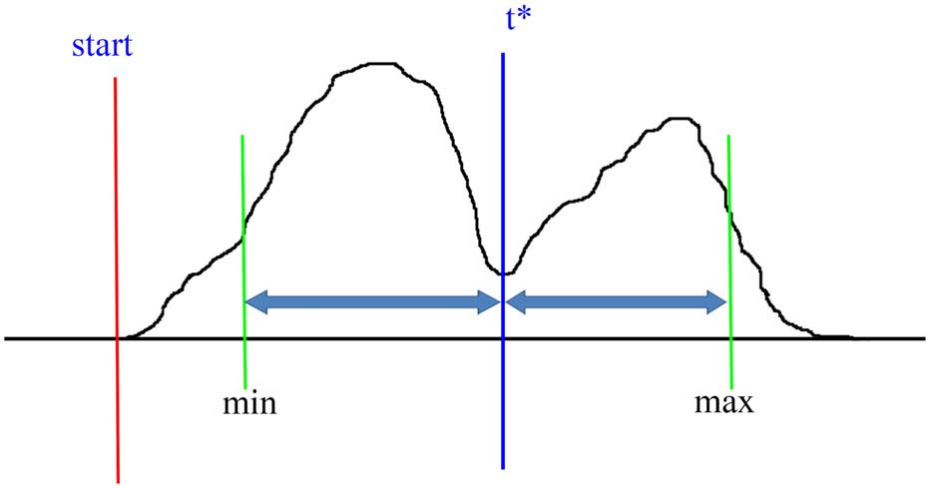
An example of finding the threshold 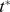 using the CMT algorithm.

From another perspective, we compared the true positive (TP) and false positive (FP) rates for each method. Figure 4 shows the ROC curve for CMT, T-PIC and MACS on the FoxA1 dataset. Also, Table 6 shows the corresponding area under curve (AUC) values. As shown in the plot and the table, CMT, again, performs better than the MACS and T-PIC.

**Table 6:** Area under curve (AUC) comparison between CMT, MACS and T-PIC, based on the number of false positive (FP) and true positive (TP) detected peaks.

	CMT	T-PIC	MACS
AUC	0.856	0.794	0.712

### Analysis of Genomic Features

**Figure 5:**
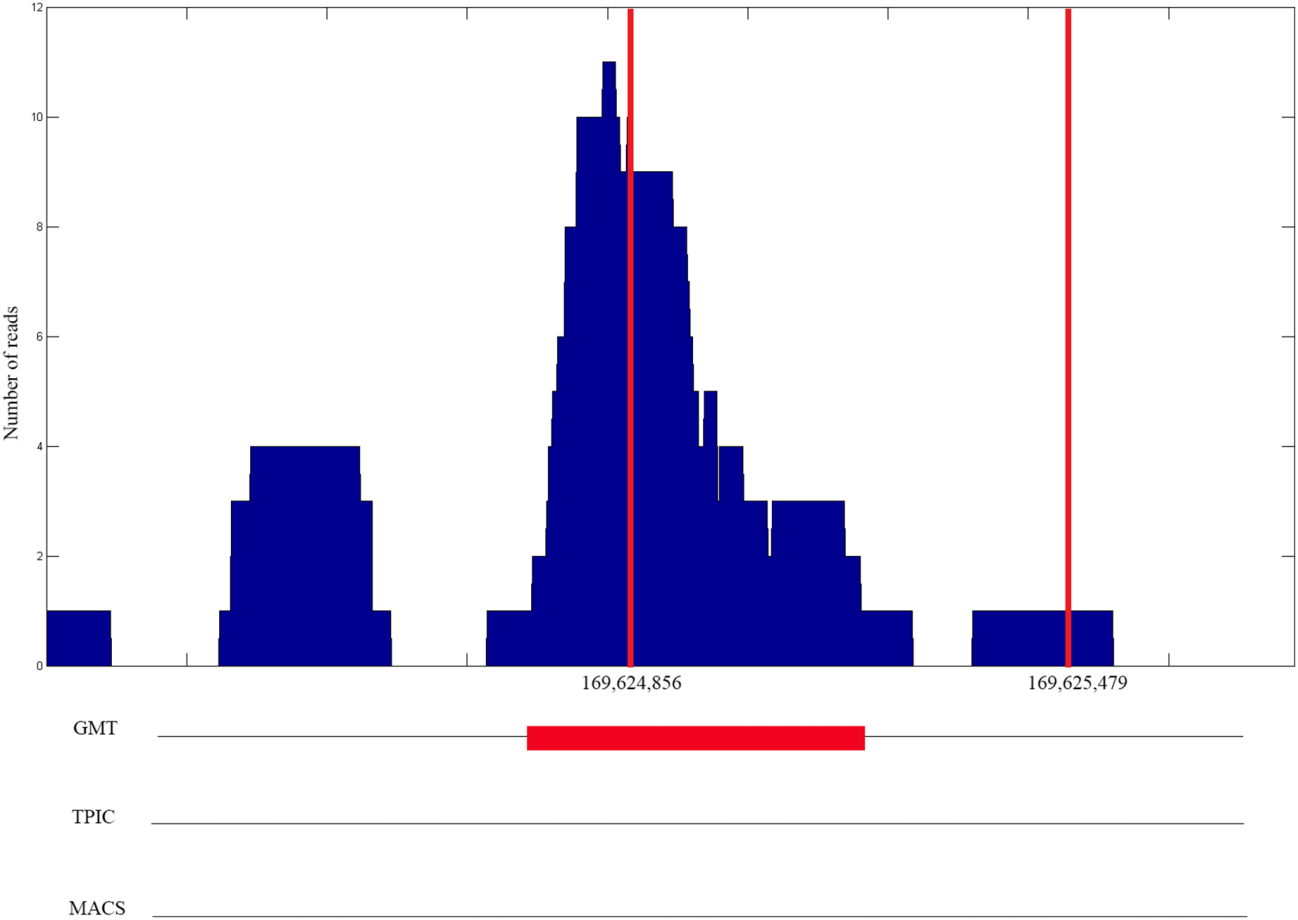
One of the true positive regions located in chromosome 3 of the FoxA1 dataset. The red lines show the actual location of the previously verified true positive region. The 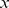-axis corresponds to the genome position in bp and the 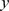-axis corresponds to the number of reads. The peak is detected by CMT but not by T-PIC or MACS.

We have also biologically validated the peaks detected by CMT on the results of independent qPCR experiments for the FoxA1 protein. We consider 25 true positives and 7 true negatives (regions) reported in [23]. The results of the other two well-known methods, T-PIC and MACS, are included in the comparison. Table 7 shows the results of this biological validation of each method. As the other two methods, CMT has been able to reject all true negatives. Although CMT finds a larger number of regions, it shows a high sensitivity, finding more true positives than T-PIC and MACS. As an example, one of the true positive regions in chromosome 3 is shown in Figure 5. The region is detected by CMT but not by T-PIC or MACS.

**Table 7:** True positive and true negative peak comparison.

	CMT	T-PIC	MACS
TP	14	13	12
TN	0	0	0

The comparison of CMT, MACS and T-PIC is based on the number of true positive (TP) and true negative (TN) detected peaks.

In another experiment, using the information gathered from the UCSC Genome Browser on the *NCBI36/hg19* assembly, the genomic features of each detected peak have been investigated. We assigned a genomic feature to a peak if that peak overlaps with the region containing that genomic feature. A detected peak can be aligned to more than one genomic feature. For example, if a specific peak overlaps with a gene and exon simultaneously, we count that peak as both gene *and* exon. Table 8 shows the percentage of regions that are located in gene, promoter, intron and exon areas as well as inter-genetic regions. CMT was able to detect more regions corresponding to genes, promoters and exons, while the percentage of regions within introns and inter-genetic areas detected by CMT is less than the percentage of detected regions by MACS and T-PIC. We have also analyzed the genomic features of the peaks detected by each method and not by the others. Table 9 shows the result of this analysis. As shown in the table, again, CMT found more genes, exons and promoters than T-PIC and MACS, while it found less peaks corresponding to the non-coding regions.

**Table 8:** Comparison of CMT, MACS and T-PIC, based on the percentage of detected regions that are associated with different genomic features.

Method	Number of Regions	Genes		Exons		Introns		Promoters		Inter-genetic Regions	
		Regions	%	Regions	%	Regions	%	Regions	%	Regions	%
MACS	14,026	12,249	87.3	967	6.9	12,438	88.7	676	4.8	7,338	52.3
T-PIC	21,662	19,041	87.9	1,721	7.9	18,731	86.5	934	4.3	10,989	50.7
CMT	26,253	23,311	88.8	2,231	8.5	22,143	84.3	1,226	4.7	13,053	49.7

**Table 9:** Comparison of CMT, MACS and T-PIC, based on the percentage of regions detected by one method and not by the others.

Method	Genes	Exons	Introns	Promoters	Inter-genetic Regions
MACS	70.5%	7.5%	71.4%	3.8%	57.4%
T-PIC	67.7%	9.8%	68.4%	2.8%	57.5%
CMT	89.1%	10.2%	68.5%	4.3%	47.2%

### Targeting a Specific Range of Regions Using Constraints

There are different types of regions of interest within the genome with various lengths. Some of the regions are long-range in the sense that have a length of up to 60 kbp such as histone modifications sites. Some other regions are mid-range such as DNA polymerase binding sites, or genes in which the length of the corresponding regions can vary from 1 to 20 kbp. There are also some regions of interest with a very small footprint such as exons of length approximately 100 bp and transcription factor binding sites of length around 10 bp.

To find a specific type of biomarker, it is better to search for regions within a certain range in the genome. Finding all regions of interest corresponding to a target protein and selecting only those regions that are wide enough to be a histone modification site or a gene increase the computational complexity of the method without adding any benefit to the analysis. Using a constraint-based model helps us target only those regions that are in a specified range. Moreover, the sensitivity of the algorithm can be adapted dynamically to target the regions of interest based on the specified range with higher accuracy.

## Methods

**Figure 6:**
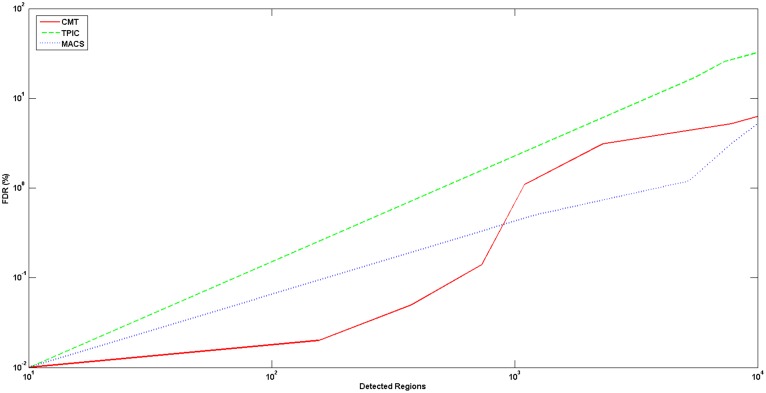
Comparison between CMT, MACS and T-PIC based on the FDR rate and number of peaks.

The aim is to find significant peaks corresponding to regions that interact with the protein of interest. Roughly speaking, each peak can be seen as a cluster that is separated from its neighbours by “valleys”. In that sense, the problem can be formulated as a *one-dimensional clustering* problem. Figure 6 depicts the process of finding the peaks corresponding to the regions of interest for the specified protein. After extending each read to a fragment, a histogram is created for each chromosome using those fragments. In the next step, relevant peaks are selected by CMT after fine tuning the exact position of the regions. Finally, by comparing each region with the corresponding region in the control histogram, the relevant peaks are selected.

### Creating the Histogram

The first step of the method consists of creating a histogram using the input BED file containing the position and direction of the reads. Each read should be extended to a fragment length, which is related to the settings used to shearing the DNA. This parameter can be input by the user, even though the fragment length can be easily estimated from the underlying data if enough computational resources are available.

After extending each read to a fragment length based on the direction of each read, each fragment is aligned to the reference genome based on its coordinates. Afterwards, for each chromosome, two separate histograms for experiment and control datasets are created for further processing. Each bin in the histogram corresponds to a nucleotide.

### The Constrained Thresholding Algorithm

For each chromosome, the corresponding experiment histogram, which is obtained from the previous step, is analyzed separately using the constraint-based algorithm. In this algorithm, each region is treated as an independent cluster. By starting from the beginning of the chromosome and based on the minimum and maximum ranges of the target regions (determined by the user), the best point to divide the histogram is found.

Although various parametric and non-parametric thresholding methods and criteria have been proposed, the three most important streams are Otsu’s method, which aims to maximize the separability of the classes measured by means of the sum of between-class variances [24], the criterion that uses information theoretic measures in order to maximize the separability of the classes [25], and the minimum error criterion [26]. In this work, we use the between-class variance criterion because it has been shown to provide very good performance in finding enriched regions in ChIP-Seq data [27] and on gridding DNA microarray images [28].

Consider a histogram 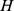, an ordered set 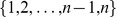, where the 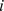
th value corresponds to the 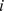
th bin and has a probability, 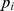. Also, consider a threshold set 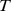, defined as an ordered set 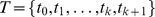, where 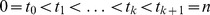 and 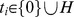. The aim of CMT is to find the values of 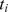 within a window starting from the current position and based on the given minimum and maximum length defined by the user.

The between class variance criterion is given by [29]:
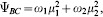
(2)where 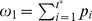, 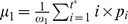, 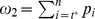 and 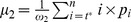.

**Figure 7:**
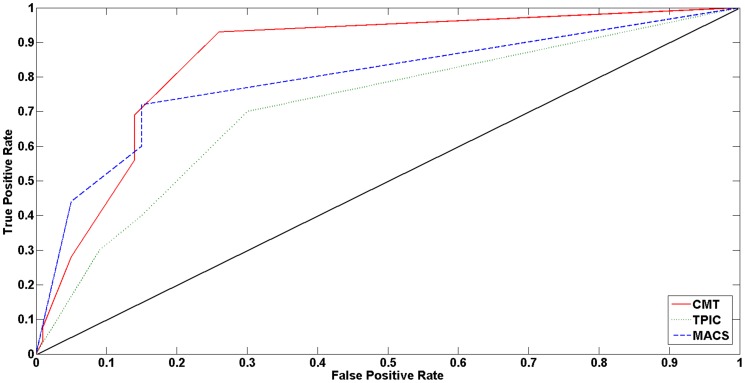
ROC curve corresponding to CMT, T-PIC and MACS.

The aim is to obtain 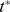 for each potential region in such a way that 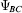 is maximized for that window. Figure 7 depicts the procedure for finding threshold 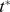. The sub-optimal threshold 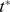 can be found by sliding the blue line between min and max and compute 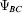 respectively. The best point to separate two neighbour peaks is the one that maximizes 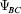.

The final output of the model consists of two vectors, 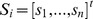 and 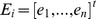, where 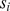 and 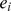 are the start and end position of the 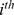 detected region respectively and 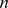 is the number of detected peaks. Although this method is not optimal, its worst-case time complexity is 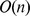, where 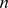 is the number of genomic positions (nucleotides) in a chromosome.

### Gap Skipping

After aligning the reads to the reference genome, and depending on the number of reads obtained from the experiment, the fragments may cover a small fraction of the genome and leave very large gaps between neighbour regions. To speed up the peak finding process, gaps are skipped by computing the maximum height of each window. If that height does not surpass the minimum acceptable height for the region, that window is skipped and no further analysis is done on the regions within that window. The minimum acceptable height is a user-adjustable value that specifies how many reads a region should support to make it acceptable as a possible region of interest.

### Selecting Enriched Regions

After finding the potential regions, they have to be shrunk from the borders for removing possible empty gaps on the left and right sides of the region. Starting from the highest point of the region, the start and end borders are moved to left and right, respectively, until the height of the region in both of those points reaches a value below a cut-off level. The cut-off level is adjustable by the user. The default value is 1, which means that the algorithm will isolate the continuous part of the region that contains at least one fragment aligned to those positions.

In the next step, the isolated experiment regions detected in the previous step are compared to their corresponding regions in the control histogram. A region in the experiment histogram is considered as an enriched region if it satisfies the following properties:

- the size of the region should be within the acceptable ranges defined by the user, and.
- there should be a *k*-fold difference between the squared density of the experiment region and the control region as follows:
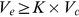
(3)where 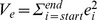, 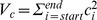; 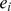 and 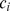 are the heights of the experiment and control regions at position 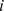, respectively. Also, 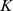 is a user-defined parameter (whose default value is 2), and corresponds to the minimum acceptable fold change between experiment and control.

The regions that satisfy the aforementioned criteria are considered enriched and are used for further processing and biological validation.

## Implementation

CMT has been implemented in C++. It runs on x86 systems using the Windows operating system. The executable version of the code is available at http://luisrueda.cs.uwindsor.ca/software/CMT-ChIP-Seq.rar. The source code is available upon request. A readme file is included in the downloadable package.
